# Influence of forest vegetation restoration on carbon increment after mining

**DOI:** 10.1038/s41598-023-45332-8

**Published:** 2023-11-10

**Authors:** Hang Zou, Yali Song

**Affiliations:** 1 Chongqing Jiaotong University, Chongqing, 400074 China; 2 Chongqing Institute of Green Intelligent Technology, Chinese Academy of Sciences, Chongqing, 401122 China; 3 Chongqing College of Chinese Academy of Sciences, Chongqing, 400714 China; 4 Department of Ecology and Environment, Southwest Forestry University, Kunming, 650224 China

**Keywords:** Ecology, Forest ecology

## Abstract

We have clarified the study area has a history of 65 years and has been restored for 6 years. This study investigated the carbon storage characteristics of undisturbed natural forests and restored mining vegetation in Yunnan Province, China. The goal was to quantify carbon reserves and increments to inform ecological restoration strategies. Four vegetation components (tree, shrub, herb, litter) and five soil layers (0–10, 10–20, 20–30, 30–40, 40–60 cm) were analyzed. In natural forest, the tree layer stored 60% of carbon (273 Mg ha^−1^), overwhelmingly dominating vegetation carbon stocks. Shrub, herb, and litter layers each comprised < 1%. Surface soil layers (0–30 cm) stored 64% of soil carbon. In the restored mining area, the tree layer contributed 75% of vegetation carbon increment (16 Mg ha^−1^), though stocks were lower than natural forest. Soil layers showed the highest carbon increment (69%) despite lower biomass than natural conditions. Unexploited forests thus exhibit robust carbon storage, while restored mining areas have weaker carbon gains, indicating recovery potential. Strategic interventions targeting soil quality, stimulating vegetation growth, and increasing carbon sequestration could significantly augment reserves and ecological functionality. Prioritizing vegetation succession and soil revitalization are paramount to ensuring ecological integrity and sustainable development. Fostering a positive regional ecological feedback loop will be pivotal. This research quantifies carbon storage differences between undisturbed and restored mining areas, highlighting soil and vegetation as critical targets for optimizing carbon sequestration and ecosystem recovery in degraded environments.

## Introduction

The carbon cycle, a crucial process within the Earth system, relies on complex material cycles and energy flows^1,2^. With the pressing global problem of climate change, the storage of carbon, especially in abandoned mining sites, is a major environmental challenge. As the world's leading producer and consumer of mineral resources, China has relied heavily on mining for economic growth and energy production, underscoring the need for solutions that reconcile economic progress with sustainable development^3^. In response, China's State Council approved the National Plan for Addressing Climate Change (2014–2020) in September 2014, which outlines a comprehensive strategy to reduce greenhouse gas emissions, promote low-carbon development and combat climate change^4^. As a high-carbon sector, mining contributes emissions throughout its lifecycle, from extraction and processing to use, as well as associated land encroachment and pollution, which hinder regional economic development^5,6^. Therefore, adopting a low-pollution, low-consumption, high-resource-use and recycling approach is imperative for the transformation and development of mining areas.

China's abundance of mineral resources has historically allowed for self-sufficiency in extraction. However, rapid advances in science and technology have led to increased demand for and extraction of minerals, exacerbating environmental challenges in mines and their surrounding areas. Negative impacts include ecological degradation, soil erosion, significant loss of biodiversity and land degradation^7,8^. To address these issues, governments have recognised the importance of abandoned mine lands and have enacted various laws and regulations to protect them^9^.

Vegetation restoration plays a critical role in enhancing the carbon stock of mining ecosystems, effectively mitigating environmental challenges in these areas. The canopy carbon stock during vegetation restoration is a key component of the ecosystem carbon cycle and provides a valid assessment of ecosystem structure and function^10,11^. In China, extensive research has been conducted on the restoration and management of abandoned mine lands, resulting in innovative engineering techniques and scientific reclamation methods, such as "Land Reclamation in Open Pit Mines" and "Mine Measurement in Mine Land Creation and Field Restoration Work"^12^. By incorporating international land reclamation and ecological restoration techniques, the efficiency of land reclamation has been greatly improved. By prioritising green mining and cultivating a low-carbon circular economy, the mining industry can mitigate the impact of production activities on the ecological environment, thereby addressing the challenges of economic development and resource environmental protection.

While previous studies have highlighted the importance of land reclamation and ecological restoration techniques in revitalising degraded mine sites, few have addressed the potential for increased carbon stocks in fully degraded mine ecosystems. The interaction between vegetation growth in forest ecosystems and the ecological conditions of mine sites is complex, with improvements in water conservation, biodiversity and soil quality dependent on varying degrees of vegetation growth^13^. Therefore, this study focuses on forest ecosystems adjacent to the mining area that have not been subject to anthropogenic disturbance, as well as those within the mining area. By examining the carbon stocks and carbon increments in the forest ecosystem of the mining area, this study seeks to provide a theoretical basis for improving soil quality, increasing carbon storage in the vegetation layer, and ultimately accelerating the ecological restoration of mining areas.

## Materials and methods

### Overview of the study area

**Figure 1:**
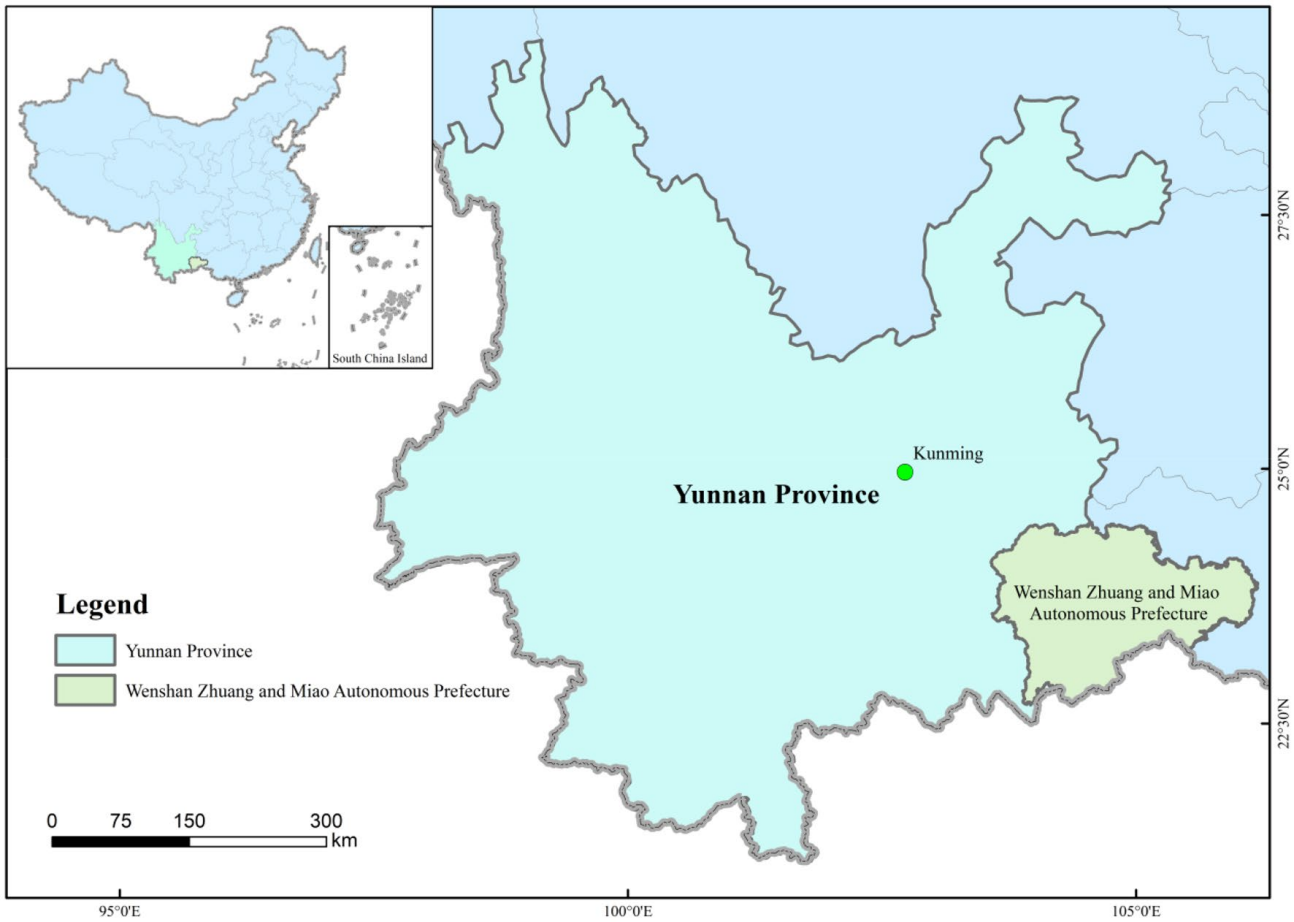
Geographic location of the mine and sampling sites.

The mining study area is located in the Shan Zhuang and Miao Autonomous Prefecture of Yunnan Province, China (Fig. 1). The roads leading to the site are national secondary and tertiary roads and are fully asphalted, allowing for easy transport. Mining operations began in the 1970s and the study site has undergone vegetation restoration. The primary vegetation types of the site include subtropical evergreen deciduous forest, mixed coniferous forest and deciduous coniferous forest dominated by fir (Cunninghamia lanceolata) and kai (Alnus cremastogyne Burk). The topography is complex, with significant height differences, low forest cover and a single species structure of timber and charcoal forest dominated by fir. Forest and grass cover in the project area is 34.24% and the primary soil-forming parent materials include limestone, Quaternary red clay and recent sediment. Soil survey data show that the project area is divided into six soil types, 12 subsoils, 16 soil genera and 19 representative soil species, dominated by red clay, red soil, limestone (rock) soil and rice soil.

### Research methodology

#### Sample plot set-up

The carbon stocks of the natural forest vegetation in the vicinity of the mining area are measured to assess the carbon storage in the natural forest vegetation of the mining area under natural conditions.The objective of the experiment was to measure carbon increment by assessing the carbon stock of forest vegetation under natural conditions around the mine site. To ensure consistency between the experimental sample plots, three standard sample plots were established, each measuring 20 m x 20 m. Factors such as climate, vegetation, topography, site conditions and mine density were considered in the selection of sample plots. In addition, five 5 m x 5 m shrub thicket plots, five 1 m x 1 m herbaceous community plots and three 1 m x 1 m litters plots were established along the diagonal of each sample plot. These were used to collect data on shrubs, herbs and litters. Table 1 shows the basic conditions of natural vegetation samples around the mining area.

**Table 1:** Sample plot layout of natural vegetation around the mining area Natural vegetation around the mining area.

No	Altitude/m	Forest type	Forest age/a	Average diameter at breast/cm	Average tree height/m	Crown width/m	Slope direction	Slope / ( )^。^
1	1250	Theropencedrymion	18	17	12	20	W108^。^	20
2	1340	Theropencedrymion	15	13	8	6	SE146^。^	15
3	1350	Subtropical evergreen broad-leaved forest	20	19	10	44	NW332^。^	12

N stands for north direction.

Three standard plots with an area of 20 m × 20 m were established within the mining area to measure the carbon increment in vegetation and soil after vegetation restoration. Along the diagonal direction of each plot, five shrub plots with an area of 5 m × 5 m and five herbaceous community plots with an area of 1 m × 1 m were set up. Additionally, three litter plots with an area of 1 m × 1 m were established to collect samples of shrubs, herbaceous plants, and litter. Table 2 shows the basic situation of vegetation restoration samples in the mining area.

**Table 2:** Layout of vegetation plots in the mining area.

No	Altitude/m	Forest type	Forest age/a	Average diameter at breast/cm	Average tree height/m	Crown width/m	Slope direction	Slope / ( )^。^
1	1250	Theropencedrymion	5	5	4	2	N353^。^	21
2	1340	Theropencedrymion	0	0	0	0	N8^。^	15
3	1340	Subtropical evergreen broad-leaved forest	8	11	8	4	N341^。^	8

#### Sample collection

All samples (natural or mine) are handled separately once they are sent to the laboratory and plant and soil samples are not mixed to avoid confusion between samples.

(1) Collection of plant samples.

Collect different organs (leaves, branches, stems, bark and roots), shrub layers (leaves, roots, branches) and herb layers (above and below ground) of typical standard trees in each sampling area. Collect 500 g of each characteristic plant part separately and weigh them in the field. At the same time, take photographs of the characteristics and growth of each plant, such as flowers, leaves, fruits, etc. Based on the Flora of China and related works and literature, accurately identify the species and genera of wild plants and conduct field surveys. Record the number of layers of trees, species names, tree heights and diameter at breast height; record the species names, number of plants (clumps) and height of layers of shrubs and herbs, and estimate the coverage; litter according to its contact distance with the ground.

The process of litter collection must be carried out carefully to maintain the integrity and accuracy of the sample. Litter is usually divided into three levels of decomposition: undecomposed layer, semi-decomposed layer and decomposed layer. Undecomposed layer usually refers to the surface litter, mainly containing leaves, twigs and other substances that have not significantly decomposed. Semi-decomposed layer is a layer between the undecomposed layer and the decomposed layer, containing some decomposed but not completely degraded litter. The decomposed layer is the layer of litter that is clearly decomposed and incorporated into the soil^14^. The following steps should be followed when collecting litter: use a small shovel or similar tool and carefully remove different decomposition layers in the soil to ensure that the samples obtained are representative. Finally, during the collection process, attention should be paid to the degree of decomposition of the litter to ensure that the samples belong to different decomposition layers.The collected materials of different decomposition layers should be placed in bags or containers marked with the sampling point information to ensure that the identification of the samples is clear and accurate. During the collection process, it is necessary to keep the sample dry and clean to avoid mixing of foreign materials. In addition, the location and date of sampling are recorded to ensure that subsequent analysis is accurate.

At the same time, the collected samples of trees, shrubs, herbs and litter were sent back to the laboratory and dried at 65 ℃ to constant weight to calculate biomass.

(2) Soil sampling.

In each sample, 5 soil profiles are excavated and 5 mixed soil samples are taken from different depths.The proportion of gravel in the soil layer below 60 cm is relatively high, and it is not in the soil layer. The soil is divided into 5 layers, namely 0–10, 10–20, 20–30, 30–40 and 40–60 cm, with approximately 500 g of soil sample collected from each layer. For each soil layer profile, a 100 cm^3^ ring knife is required to take soil samples, and the soil samples are dried at a temperature of 105 ℃ before the bulk density is measured. At the same time, each soil layer is sampled separately.

The sample is cleaned of small debris and other contaminants and then returned to the laboratory for natural air drying. The soil sample is then ground and sieved (100 mesh) for the determination of soil organic carbon^15^.

#### Calculation of carbon content and carbon increment of sample plots

The carbon storage of natural forest vegetation in the mining area was measured based on the carbon storage of forest vegetation under natural conditions surrounding the mining area. The carbon increment in the mining area was measured based on the carbon storage of vegetation and soil after vegetation restoration in the mining area. Determination of organic carbon content in plant and soil samples using potassium dichromate volumetric method in the laboratory^16^:

1 $${\text{VCS}} = {\text{OC}} \times {\text{B}}$$

In the equation, VCS represents the carbon storage and carbon increment of the vegetation layer. (Mg ha^−1^), OC is the organic carbon content (%) and B is the biomass per unit area (Mg ha^−1^).

2 $${\text{TN}} = \sum {\text{CN}} \times {\text{PN}} \times {\text{DN}}$$

In the equation, TN represents the total soil carbon storage and carbon increment within the depth profile of the soil layer. (g∙cm^−3^) in the layer soil profile depth; CN is the soil carbon content (%) in layer N; PN is the soil capacity (g∙cm^−3^) in layer N: DN is the layer N profile depth.

#### Analytical processing of data

One-way ANOVA was used to test the variability of carbon increments in each stand, and Excel 2013 and SPSS 20.0 were used to process the graphs and carry out the data analysis.The map in Fig. 1 was generated using ArcGIS Desktop 10.7 (Esri, Redlands, CA, USA). ArcGIS is a geographic information system (GIS) software that allows for mapping and spatial analysis of data. Version 10.7 was used to create the map by importing and georeferencing the relevant datasets, applying symbology, and exporting the map as an image file for inclusion in the article. The latest version of ArcGIS Desktop can be downloaded from https://www.esri.com/en-us/arcgis/products/arcgis-desktop/overview.

## Results and discussion

### Carbon stock characteristics of natural vegetation in the mining area

#### Carbon content, biomass and carbon stock characteristics of the tree layer

According to Table 3, the carbon content in the tree layer shows the following order: stem > leaf > bark > root > branch, ranging from 48.05% to 58.76%. The biomass of the tree layer is highest in the stem (198.76 Mg ha^−1^), followed by the branch (143.04 Mg ha^−1^) and the root (73.12 Mg ha^−1^), while the leaf (51.21 Mg ha^−1^) and the bark (28.28 Mg ha^−1^) have the lowest biomass. Carbon storage in the stem accounts for the majority of carbon storage in the tree layer, representing 45% of the total, followed by the branch (25%) and root (12%), while the leaf (10%) and bark (5%) have the smallest contributions. The order of carbon storage is stem > branch > root > leaf > bark.

**Table 3:** Tree layer carbon content, biomass and carbon loss reserves.

Components	ω (C)/%	Biomass/(Mg ha^-1^)	Carbon stock (Mg ha^-1^)
Leaf	55.46 ± 3.78a	51.21 ± 16.24b	27.63 ± 4.07c
Branch	48.05 ± 1.10a	143.04 ± 37.68a	70.67 ± 7.10b
Stem	58.76 ± 3.17a	198.76 ± 51.05a	123.84 ± 25.03a
Bark	53.28 ± 3.53a	28.28 ± 7.28c	15.76 ± 3.17c
Root	51.21 ± 7.94a	73.12 ± 23.63b	35.20 ± 4.64c
Average/Total	53.29 ± 3.91	494.42 ± 135.89	273.12 ± 44.03

In the "Mean/total" column for components, carbon content is the mean, and biomass and carbon increment are total. Different lowercase letters in the same column indicate significant differences (*P* < 0.05); mean ± standard deviation, n = 3. Same below.

### Carbon content, biomass and carbon stock characteristics of shrub layer

Based on the carbon content of the shrub layer (Table 4), the order from highest to lowest is branch > root > leaf. Among the organs of the shrub layer, there is no significant difference between the root and the branch (*P* > 0.05), while the leaf shows a significant difference from the root and the branch (*P* < 0.05). The biomass distribution of the different organs in the shrub layer is consistent with the carbon content. The branch has the highest biomass in the shrub layer with 44.7% of the total biomass, followed by the root (34.7%) and the leaf has the lowest biomass (20.6%). The order is branch > root > leaf.

**Table 4:** Carbon content, biomass and carbon loss of shrub layer.

Components	ω (C)/%	Biomass/(Mg ha^-1^)	Carbon stock (Mg ha^-1^)
Leaf	29.03 ± 4.47b	11.34 ± 0.80b	1.41 ± 0.18b
Root	41.60 ± 2.64a	19.27 ± 1.78a	2.84 ± 1.03b
Branch	48.14 ± 4.84a	24.77 ± 2.35a	4.99 ± 0.53a
Average/Total	39.59 ± 9.13	55.38 ± 6.04	9.24 ± 1.66

When comparing different vegetation types within the same shrub layer, there are differences in biomass and significant differences between organs (*P* < 0.05). The main organ contributing to the carbon storage in the shrub layer is the branch, which accounts for 54.0% of the carbon storage in the shrub layer, followed by the root (30.7%), and the leaf has the smallest contribution (16.3%). The order is: branch > root > leaf.

### Carbon content, biomass and carbon stock characteristics of the herbaceous layer

The below ground part of the herbaceous layer accounts for 55.1% of the carbon content, followed by the above ground part (44.9%). The carbon content is higher in the belowground part than in the aboveground part (Table 5). The biomass of the aboveground part of the herbaceous layer (10.15 Mg ha^−1^) is higher than that of the belowground part (7.10 Mg ha^−1^). Similarly, the above-ground portion contributes to 57.6% of the carbon storage in the herbaceous layer, followed by the below-ground portion (42.4%).

**Table 5:** Carbon content, biomass and carbon storage of herbaceous layer.

Components	ω (C)/%	Biomass/(Mg ha^-1^)	Carbon stock (Mg ha^-1^)
Aboveground	21.99 ± 5.08a	10.15 ± 2.03a	1.65 ± 0.38a
Underground	27.00 ± 6.64a	7.10 ± 4.15a	1.21 ± 0.23a
Average/Total	24.45 ± 5.86	17.25 ± 6.18	2.86 ± 0.61

### Carbon content, biomass and carbon stock characteristics of the litters layer

The carbon content in the litter layer shows a significant difference (*P* < 0.05) between the different decomposition stages, with the undecomposed layer having the highest carbon content, followed by the partially decomposed layer and the fully decomposed layer. The carbon content decreases with increasing degree of decomposition (Table 6). The biomass distribution in the litter layer is highest in the fully decomposed layer (6.96 Mg ha^−1^), followed by the partially decomposed layer (3.59 Mg ha^−1^), and lowest in the undecomposed layer (1.24 Mg ha^−1^), indicating a pattern of fully decomposed layer > partially decomposed layer > undecomposed layer, which shows significant differences (*P* < 0.05). In terms of carbon storage in the litter layer, the partially decomposed layer has the highest carbon storage, accounting for 63.9% of the carbon storage in the litter layer, followed by the partially decomposed layer (24.5%), and the fully decomposed layer has the lowest carbon storage (11.6%).

**Table 6:** Carbon content, biomass and carbon loss reserves of litter layer.

Components	ω (C)/%	Biomass/(Mg ha^-1^)	Carbon stock (Mg ha^-1^)
Fresh litter layer	39.18 ± 3.73a	1.24 ± 0.61b	0.79 ± 0.23b
Fragmented litter layer	19.84 ± 4.57b	3.59 ± 1.36b	2.06 ± 0.55a
Humified litter layer	9.90 ± 1.84b	6.96 ± 1.50a	0.36 ± 0.13b
Average/Total	22.97 ± 5.07	11.80 ± 3.49	3.22 ± 0.93

### Carbon content, biomass and carbon stock characteristics of soil layers

The soil carbon content at the three sampling sites decreases significantly with increasing soil depth (*P* < 0.05). The distribution of soil carbon content varies between different soil layers, with the topsoil layer (0^–1^0 cm) having the highest carbon content and the 40–60 cm soil layer having the lowest carbon content (Table 7). The factors influencing soil carbon storage are soil carbon content and bulk density. As soil carbon storage increases, soil depth decreases and the magnitude of the change is generally consistent with soil carbon content. Soil carbon storage in the 0–30 cm soil layer accounts for 64.2% of total soil carbon storage, indicating that the majority of soil carbon storage is concentrated in the surface soil layer.

**Table 7:** Carbon content, biomass and carbon loss characteristics of soil layer.

Soil layer/cm	ω (C)/%	Biomass/(Mg ha^-1^)	Carbon stock (Mg ha^-1^)
0–10	24.12 ± 5.15a	0.67 ± 0.11b	44.1 ± 6.3a
10–20	18.04 ± 5.49b	1.55 ± 0.31b	36.6 ± 8.0a
20–30	14.36 ± 1.23b	2.52 ± 0.16a	28.5 ± 9.3b
30–40	9.39 ± 1.06b	2.89 ± 0.17a	21.5 ± 5.0b
40–60	3.81 ± 1.10c	3.24 ± 0.38a	39.0 ± 21.0a
Average/Total	13.94 ± 2.80	10.88 ± 1.15	169.9 ± 12.7

### Carbon content, biomass and carbon stock characteristics of natural vegetation

The carbon content of the different components in natural vegetation follows the order: tree layer > soil layer > litter layer > herb layer > shrub layer (Table 8). In natural vegetation, the main carbon storage component is the tree layer, which accounts for 60% of the carbon storage in natural vegetation. The soil layer contributes 37% of the carbon storage, while the shrub layer (0.6%), the herbaceous layer (0.6%) and the litter layer (0.7%) have relatively small shares in carbon storage.

**Table 8:** Carbon content, biomass and carbon loss reserves of natural vegetation.

Components	ω (C)/%	Biomass/(Mg ha^-1^) /Bulk density/(g/cm^3^)	Carbon stock (Mg ha^-1^)
Arbor layer	53.29 ± 3.91a	494.42 ± 135.89a	273.12 ± 44.03a
Shrub layer	39.59 ± 9.13b	55.38 ± 6.04b	9.24 ± 1.66b
Herb layer	24.45 ± 5.86b	17.25 ± 6.18c	2.86 ± 0.61b
Litter layer	22.97 ± 5.07b	11.80 ± 3.49c	3.22 ± 0.93b
Soil layer	13.94 ± 2.80c	10.88 ± 1.15c	169.9 ± 12.7a

## Characteristics of the carbon increment of vegetation restoration in mining areas

### Carbon content, biomass and carbon increment characteristics of the tree layer

Table 9 shows that within the tree layer the carbon content followed a descending order: leaf > root > branch > bark > stem. The highest biomass in the tree layer was found in roots (45.00 Mg ha^−1^), followed by leaves (40.91 Mg ha^−1^) and stems (31.33 Mg ha^−1^), while the lowest biomass was found in peels (29.66 Mg ha^−1^) and branches (26.36 Mg ha^−1^). Leaves were responsible for the largest carbon increment in the tree layer, contributing 47.3% of the total, followed by roots (25.1%) and branches (11.6%). The smallest carbon increment came from the stem (8.8%) and bark (7.2%). In summary, the order of carbon accumulation in the tree layer was: leaves > roots > branches > stem > bark.

**Table 9:** Carbon content, biomass and carbon increment in the tree layer.

Grouping	Carbon content/%	Biomass/(Mg ha^-1^)	Carbon gain (Mg ha^-1^)
Leaf	18.97 ± 16.72c	40.91 ± 35.61b	7.76 ± 5.95c
Branch	7.25 ± 7.65b	26.36 ± 27.75a	1.91 ± 0.42a
Dry Stem	4.63 ± 4.01a	31.33 ± 29.20a	1.45 ± 0.50a
Pi Bark	4.89 ± 4.35a	29.66 ± 27.06a	1.13 ± 0.23a
Root Root	9.19 ± 9.12b	45.00 ± 15.00b	4.13 ± 0.71b
Average/Total	8.99 ± 8.37	161.58 ± 149.71	16.39 ± 2.64

### Carbon content, biomass and carbon increment characteristics of the shrub layer

As for the shrub layer (Table 10), branches had the highest carbon content, followed by roots and leaves, which differed significantly from each other (*P* < 0.05). Branches also accounted for the largest part of the shrub layer biomass (43.3%), followed by leaves (29.5%) and roots (29.2%). The biomass distribution of the shrub layer organs differed significantly (*P* < 0.05), in the order: branches > leaves > roots. Branches were also responsible for the highest carbon increment in the shrub layer, contributing 59.1% of the total, followed by roots (24.3%) and leaves (16.6%). Therefore the order of carbon increment in the shrub layer was: branches > roots > leaves.

**Table 10:** Carbon content, biomass and carbon increment in the shrub layer.

Grouping	Carbon content/%	Biomass/(Mg ha^-1^)	Carbon gain (Mg ha^-1^)
Leaf	9.26 ± 4.19a	2.06 ± 1.85a	0.19 ± 0.07a
Root Root	15.80 ± 3.37b	1.80 ± 0.62a	0.28 ± 0.09a
Branch	22.49 ± 3.00b	3.03 ± 0.51b	0.68 ± 0.04b
Average/Total	15.85 ± 3.52	6.89 ± 2.99	1.15 ± 0.20

### Carbon content, biomass and carbon increment characteristics of the herbaceous layer

In the herbaceous layer (Table 11), the carbon content was higher in the above-ground part than in the below-ground part, with shares of 55.3% and 44.7%, respectively. However, biomass was higher in the below-ground part (0.54 Mg ha^−1^) than in the above-ground part (0.26 Mg ha^−1^). Similarly, the carbon increment in the below-ground part accounted for 66.7% of the herbaceous layer, while the above-ground part contributed 33.3%.

**Table 11:** Carbon content, biomass and carbon gain in the herbaceous layer.

Grouping	Carbon content/%	Biomass/(Mg ha^-1^)	Carbon gain (Mg ha^-1^)
Above ground section	5.97 ± 2.70a	0.26 ± 0.55a	0.01 ± 0.03 a
Ground floor section	4.83 ± 2.55a	0.54 ± 0.11a	0.02 ± 0.02a
Average/Total	5.40 ± 2.55	0.80 ± 0.66	0.03 ± 0.05

### Characteristics of carbon content, biomass and carbon increment in the litters layer

Regarding the litters layer (Table 12), the carbon content decreased with the decomposition of the material in the following order: undecomposed layer > semi-decomposed layer > decomposed layer. The carbon content of the different organs also differed significantly (*P* < 0.05). The biomass distribution of the litters layer was opposite to the carbon content, with the highest biomass found in the decomposed layer (1.76 Mg ha^−1^), followed by the semi-decomposed layer (1.22 Mg ha^−1^) and the lowest biomass in the undecomposed layer (0.98 Mg ha^−1^). In terms of carbon increment, the highest contribution came from the decomposed layer (40.0%), followed by the semi-decomposed layer (31.4%), and the lowest from the undecomposed layer (28.6%). Therefore, the order of carbon increment in the litters layer was: decomposed layer > semi-decomposed layer > undecomposed layer.

**Table 12:** Carbon content, biomass and carbon increment in the litters layer.

Grouping	Carbon content/%	Biomass/(Mg ha^-1^)	Carbon gain (Mg ha^-1^)
Undecomposed layer	10.94 ± 1.99a	0.98 ± 0.30a	0.10 ± 0.06a
Semi-decomposed layer	9.51 ± 1.78a	1.22 ± 0.35a	0.11 ± 0.07a
Decomposed layers	8.13 ± 1.27a	1.76 ± 0.80b	0.14 ± 0.02a
Average/Total	9.52 ± 1.53	3.96 ± 1.45	0.35 ± 0.15

### Soil layer carbon content, biomass and carbon increment characteristics

Soil carbon content was found to be strongly influenced by soil depth, with a significant decrease (*P* < 0.05) observed with increasing depth. Carbon content was unevenly distributed among the soil layers, with the highest concentration found in the topsoil layer (0–10 cm) and the lowest in the 40–60 cm layer (Table 13). In contrast, soil bulk density increased with depth, and the bulk density of the 40–60 cm layer was 3.8 times higher than that of the 0–10 cm layer.

**Table 13:** Soil layer carbon content, biomass and carbon increment.

Soil layer/cm	Carbon content/%	Biomass/(Mg ha^-1^)	Carbon gain (Mg ha^-1^)
0–10	13.71 ± 3.26a	0.23 ± 0.09b	13.9 ± 2.0a
10–20	7.04 ± 2.65b	0.42 ± 0.23b	11.2 ± 1.2a
20–30	3.39 ± 2.15b	0.61 ± 0.32a	8.5 ± 0.8b
30–40	6.13 ± 5.83b	0.81 ± 0.50a	6.3 ± 2.5b
40–60	1.19 ± 0.48c	0.88 ± 0.36c	8.4 ± 3.8a
Average/Total	6.29 ± 2.88	2.95 ± 1.50	3.61 ± 3.3

Both soil carbon content and capacity affect soil carbon increment, which also decreases with increasing soil depth, consistent with the trend observed for soil carbon content. The top 30 cm of soil accounted for 69.5% of total soil carbon increment, indicating that most of the carbon increment in the soil layer was concentrated in the topsoil.

### Ecosystem carbon content, biomass and carbon increment characteristics

In the vegetation layer, carbon increment is determined by both the carbon content and biomass of each component, with higher carbon content and biomass leading to higher carbon increment. In the forest ecosystem, the carbon content of each component followed the order tree layer > soil layer > litters layer > herb layer > shrub layer (Table 14). Biomass (capacity) was highest in the tree layer (161.58 Mg ha^−1^), followed by the shrub layer (6.89 Mg ha^−1^), coppice layer (0.35 Mg ha^−1^), soil layer (2.95 Mg ha^−1^) and herb layer (0.03 Mg ha^−1^). The lowest carbon accumulation in the forest ecosystem was observed in the soil layer (2.95 Mg ha^−1^) and herb layer (0.03 Mg ha^−1^), indicating that the order of carbon accumulation was tree layer > shrub layer > litters layer > soil layer > herb layer. The tree layer was found to be the main contributor to carbon increment in the forest ecosystem, accounting for 75.1% of the total, followed by the soil layer (16.5%), shrub layer (5.2%) and litters layer (1.6%), with the herbaceous layer contributing the least (0.01%).

**Table 14:** Ecosystem carbon content, biomass and carbon increment.

Grouping	Carbon content/%	Biomass/(Mg ha^-1^)	Carbon gain/(Mg ha^-1^)
Tree layer	8.99 ± 8.37a	161.58 ± 149.71d	16.39 ± 2.64d
Shrub layer	15.85 ± 3.52c	6.89 ± 2.99c	1.15 ± 0.20b
Herbaceous layer	5.40 ± 2.55a	0.80 ± 0.66a	0.03 ± 0.01a
litters layer	9.52 ± 1.53b	3.96 ± 1.45b	0.35 ± 0.15b
Soil layer	6.29 ± 2.88a	2.95 ± 1.50b	3.61 ± 3.3c

## Discussion

### Carbon stock characteristics of natural vegetation in the mining area

This study focused on estimating the carbon stock of natural vegetation in mining areas. We selected representative undisturbed natural vegetation near mining sites to investigate the carbon storage within the area.

The natural vegetation was categorized into four components: the tree layer, shrub layer, herb layer, and litter layer^17^. Carbon content varied among these vegetation types, and we determined the carbon storage by measuring carbon content and biomass, which improved the accuracy of our calculations. Biomass was the primary form of carbon accumulation in natural vegetation, influenced by regional climate, soil types, forest vegetation, and plant age^18^. Carbon storage in the tree layer dominated the overall carbon storage in the vegetation layer of the mining area, accounting for 94% of the vegetation layer and 60% of all natural vegetation. However, carbon loss from vegetation was influenced not only by vegetation composition and tree age but also by factors such as regional climate, ambient light, surface types, and cavity composition^19^. Within the tree layer, carbon storage followed the order: roots > branches > stems > leaves > bark, with leaves and bark having lower carbon storage due to tissue senescence and slower carbon cycling.

The shrub layer contributed 3% of the carbon stored in natural vegetation, significantly more than the herb and litter layers. In the herb layer, aboveground carbon storage exceeded belowground carbon storage. The litter layer played an important role in the carbon input from the forest to the soil^20,21^, with the partially decomposed layer having the highest carbon storage among the different litter components.The soil organic carbon content varied among the different soil layers, with the topsoil (0–10 cm) having the highest content and the 40–60 cm layer having the lowest. Long-term use of the mining area resulted in severe damage to vegetation and soil layers. In an undisturbed scenario, soil carbon storage would have been higher due to the presence of shrubs and litter layers, which contribute to carbon replenishment through decomposition. In addition, slow decomposition of organic matter and the presence of rich community vegetation played a role in maintaining higher soil carbon storage in the 0–30 cm soil layer, which accounted for 64% of total soil carbon storage.

The main factors influencing carbon storage in natural vegetation in mining areas were plot type, vegetation density and forest self-regulation practices. The forest ecosystem in the mining area showed higher carbon storage compared to the average of Chinese forest ecosystems (258.8 Mg ha^−1^)^22^, indicating a strong carbon sequestration potential. Therefore, it is essential to implement protection measures, improve soil quality and increase carbon reserves in the vegetation layer to restore the ecological integrity and land productivity of degraded mining areas.

### Characteristics of carbon increment in vegetation restoration in mining areas

Mining activities have brought both benefits and negative environmental impacts, such as soil erosion and carbon loss. To address these issues, this study aimed to determine the carbon increment following vegetation restoration in mining areas^23^. We selected an undisturbed forest ecosystem adjacent to the mining site as a research object to guide the selection of tree species and ecological restoration models for the mine site. Carbon content varies between different organ types and we used measured carbon content and biomass to more accurately calculate carbon increment. Biomass accumulation is the primary form of plant growth in forest ecosystems and is influenced by regional climate, soil type, forest vegetation type and plant age^24^. Biomass in the mining area was significantly lower than that in undisturbed natural forest ecosystems (552.8 Mg ha^−1^), indicating significant potential for improvement.

Vegetation recovery and succession are complex processes influenced by vegetation-soil interactions, with different vegetation types having different biomass and carbon content due to their physiological and ecological characteristics^25,26^. Soil properties also play an important role in soil organic carbon cycling. The distribution of carbon increment differs among the organic matter types, with the soil layer showing the highest carbon increment, followed by the tree layer, shrub layer, litters layer and herb layer. Studies consistently show that organic carbon content decreases significantly with increasing soil depth^27^. In our study, carbon increment in the 0–30 cm soil layer accounted for 69.3% of total soil carbon increment^28,29^, exceeding the global average for total soil carbon increment in all soil surface layers. This suggests that carbon increment in the surface layer was the major contributor to soil carbon increment in our experiment.

Sample site type, vegetation density, and forest self-management practices were critical factors influencing carbon increment in forest ecosystems^30^. Restoring the ecosystem in mining areas can significantly increase carbon increment, especially in areas with low vegetation density and degraded soil quality due to mining activities^31^. Our analysis revealed a strong positive correlation between vegetation and soil carbon stocks with subsurface biomass and soil content. Thus, targeted measures can be implemented to enhance soil factors, promote vegetation growth, increase soil and vegetation carbon stocks, and improve forest ecosystems in mining areas.

## Conclusions

1. In natural forest, trees accounted for 94.6% of biomass and 60% of carbon storage. Shrubs, herbs, and litter each comprised < 1% of carbon reserves. For soil, surface layers stored more carbon than deeper layers.
2. In restored mining vegetation, trees contributed 75.1% of carbon increments, though biomass was lower than natural forest. The soil layer had the highest carbon increment overall (69.5%), demonstrating the importance of soil restoration. Shrub and litter increments were also lower than natural conditions.
3. Undisturbed natural forests have robust carbon storage capacities, while reclaimed mining areas show lower carbon gains, indicating potential for recovery. Strategic interventions targeting soil quality, vegetation growth and carbon sequestration can enhance reserves and ecological functionality. Prioritising succession and soil revitalisation are key objectives. Through these measures, we can ensure the ecological integrity of the mining area and its surroundings, promote sustainable economic development and contribute to a harmonious society. At the same time, these efforts play a key role in creating a positive feedback loop for the regional ecological environment.

### Ethical approval

The authors and I declare that the methods used for collecting plant samples, handling and processing samples, and performing chemical experiments were in accordance with relevant institutional, national, and international guidelines, regulations, and laws. All methods were carried out in accordance with the applicable guidelines and there were no instances of malpractice or non-compliance. In addition, appropriate literature references have been provided to support the methods used.

## Data Availability

The data that support our research findings are available from the corresponding author on request.
